# Retraction: Inhibition of tumor vasculogenic mimicry and prolongation of host survival in highly aggressive gallbladder cancers by Norcantharidin via blocking the ephrin type a receptor 2/focal adhesion kinase/paxillin signaling pathway

**DOI:** 10.1371/journal.pone.0319593

**Published:** 2025-02-20

**Authors:** The PLOS One Editors

After this article [1] was published, concerns were raised regarding results presented in Figs 2, 3, 6, and 7.

Specifically

- The following panels appear to partially overlap despite representing different experimental conditions:
  Fig 2A TIMP-2 panel and NCTD panel flipped horizontally
  Fig 2B TIMP-2 panel and NCTD panel flipped vertically
  Fig 3 Control 1d, TIMP-2 1d, and NCTD 1d panels
  Fig 3 TIMP-2 1w panel and Control 2w panel flipped vertically
  Fig 3 NCTD 1w and NCTD 2w panels
- Fig 7A EphA2 Control panel: when color levels are adjusted, there appear to be regions where the background signal does not match the surrounding areas
- The following results appear similar despite representing different experimental conditions:
  ◦ Fig 7A TIMP-2 EphA2 panel of [1] and Fig 9 TIMP-2 MM1-MMP panel of [2] rotated 90 degrees
  ◦ Fig 7A NCTD EphA2 panel of [1] and Fig 9 NCTD MM1-MMP panel of [2] flipped vertically
  ◦ Fig 7A Control FAK panel of [1] and Fig 9 Control MMP-2 panel of [2] rotated 90 degrees
  ◦ Fig 7A NCTD FAK panel in [1] and Fig 9 NCTD MMP-2 panel of [2] flipped horizontally
  ◦ Fig 7A TIMP-2 Paxillin panel of [1] and Fig 9 Control MM1-MMP panel of [2]
  ◦ Fig 7B EphA2 panel of [1] and Fig 10A MM1-MMP panel of [2] flipped vertically and with altered contrast
  ◦ Fig 7B Paxillin-P panel of [1] and Fig 10A MMP-2 panel of [2]
  ◦ Fig 7B β-actin panel of [1] and Fig 10A β-actin of [2]
  ◦ Fig 7B β-actin panel of [1]: lanes 1 and 2 appear similar to lanes 5 and 6
- The images in Fig 6B of [1] appear similar to images in Fig 3A of [2] which appear to represent the same experimental conditions
- Fig 2C Control panel of [1] appears similar to Fig 3B b3 GBC-SD panel in a later article [3]

The authors did not respond to editorial requests for a response and underlying data. In light of the above unresolved concerns that question the integrity and reliability of the reported results and conclusions, the *PLOS One* Editors retract this article.

All authors either did not respond directly or could not be reached.
